# Anaerobic Nitrogen Turnover by Sinking Diatom Aggregates at Varying Ambient Oxygen Levels

**DOI:** 10.3389/fmicb.2016.00098

**Published:** 2016-02-05

**Authors:** Peter Stief, Anja Kamp, Bo Thamdrup, Ronnie N. Glud

**Affiliations:** ^1^Department of Biology and Nordic Center for Earth Evolution, University of Southern DenmarkOdense, Denmark; ^2^AIAS, Aarhus Institute of Advanced Studies, Aarhus UniversityAarhus, Denmark

**Keywords:** marine snow, sinking diatom aggregates, fixed-nitrogen loss, low-oxygen environments, intracellular nitrate, microsensors, stable isotopes

## Abstract

In the world’s oceans, even relatively low oxygen levels inhibit anaerobic nitrogen cycling by free-living microbes. Sinking organic aggregates, however, might provide oxygen-depleted microbial hotspots in otherwise oxygenated surface waters. Here, we show that sinking diatom aggregates can host anaerobic nitrogen cycling at ambient oxygen levels well above the hypoxic threshold. Aggregates were produced from the ubiquitous diatom *Skeletonema marinoi* and the natural microbial community of seawater. Microsensor profiling through the center of sinking aggregates revealed internal anoxia at ambient 40% air saturation (∼100 μmol O_2_ L^-1^) and below. Accordingly, anaerobic nitrate turnover inside the aggregates was evident within this range of ambient oxygen levels. In incubations with ^15^N-labeled nitrate, individual *Skeletonema* aggregates produced NO_2_^-^ (up to 10.7 nmol N h^-1^ per aggregate), N_2_ (up to 7.1 nmol N h^-1^), NH_4_^+^ (up to 2.0 nmol N h^-1^), and N_2_O (up to 0.2 nmol N h^-1^). Intriguingly, nitrate stored inside the diatom cells served as an additional, internal nitrate source for dinitrogen production, which may partially uncouple anaerobic nitrate turnover by diatom aggregates from direct ambient nitrate supply. Sinking diatom aggregates can contribute directly to fixed-nitrogen loss in low-oxygen environments in the ocean and vastly expand the ocean volume in which anaerobic nitrogen turnover is possible, despite relatively high ambient oxygen levels. Depending on the extent of intracellular nitrate consumption during the sinking process, diatom aggregates may also be involved in the long-distance export of nitrate to the deep ocean.

## Introduction

Marine snow comprises a variety of organic aggregates, larger than 500 μm in diameter, that are suspended in the ocean’s water column (Simon et al., 2002; Turner, 2015). Often formed in the photic surface layers, such aggregates sink down toward the seafloor and thereby mediate organic carbon export to the deep ocean (Shanks and Trent, 1980). Diatom aggregates that form in the wake of phytoplankton blooms represent a major component of marine snow and promote the mass sinking of algal biomass (Smetacek, 1985; Alldredge and Gotschalk, 1989; Thornton, 2002). Sinking aggregates also represent nutrient oases for pelagic microorganisms and thus are highly enriched in bacteria and protists compared to ambient water (Ploug et al., 1999; Grossart et al., 2003; Thiele et al., 2015). The plume of metabolites leaking out of sinking aggregates has a much larger diameter than the aggregate itself and thereby also affects the behavior and metabolism of diverse free-living organisms (Kiørboe et al., 2001; Azam and Malfatti, 2007; Stocker et al., 2008).

The individual aggregate represents a distinctive chemical microenvironment in the water column, often enriched in nutrients (Shanks and Trent, 1979; Kaltenböck and Herndl, 1992) and depleted in O_2_ (Alldredge and Cohen, 1987; Ploug et al., 1997). This microenvironment may trigger microbial processes that would otherwise not occur in the water column (Shanks and Reeder, 1993; Karl and Tilbrook, 1994). An oxygen-depleted (anoxic) center may develop inside sinking aggregates due to the respiration activity of the inhabiting microbial community. This phenomenon is more likely to occur in large aggregates (due to diffusion limitation) and at high respiration rates (due to organic matter reactivity; Jørgensen, 1977). Accordingly, anoxic centers have been directly traced with O_2_ microsensors only in relatively large organic aggregates, such as fecal pellets (Alldredge and Cohen, 1987), laboratory-made aggregates (Ploug et al., 1997; Ploug and Bergkvist, 2015), zooplankton carcasses (Glud et al., 2015), and cyanobacterial colonies (Paerl and Bebout, 1988; Klawonn et al., 2015). Even in such aggregates, anoxia may be short-lived due to carbon limitation (Ploug et al., 1997), the distribution of O_2_ may be patchy (Paerl and Bebout, 1988), and low-oxygen conditions may quickly alternate with high-oxygen conditions in photosynthetically active aggregates (Alldredge and Cohen, 1987). Conversely, at low ambient O_2_ levels, e.g., in hypoxic coastal regions and oceanic oxygen minimum zones (OMZs), smaller aggregate sizes and/or lower respiration rates are sufficient to promote the development of an anoxic center in sinking aggregates. Hence, a higher abundance of hypoxic and anoxic aggregates can be assumed to occur in low-oxygen than in high-oxygen environments (Ploug et al., 1997; Ploug, 2001; Klawonn et al., 2015).

The currently expanding low-oxygen environments in the ocean (Diaz and Rosenberg, 2008) are estimated to be responsible for 30–50% of the total oceanic fixed-nitrogen loss (DeVries et al., 2013). Sinking aggregates potentially increase the ocean volume in which fixed-nitrogen loss can occur even further. Nevertheless, sinking aggregates have still not been shown to directly contribute to this process, even though they are often suspected of providing suitable microenvironments for anaerobic nitrogen cycling (Jensen et al., 2011; Kalvelage et al., 2011; Dalsgaard et al., 2014). We hypothesize that the globally abundant diatom aggregates (Smetacek, 1985; Alldredge and Gotschalk, 1989; Kiørboe et al., 1998; Thornton, 2002; Villareal et al., 2011; Kemp and Villareal, 2013) host anaerobic nitrogen-cycle activities at ambient O_2_ levels that inhibit such activities in free-living microorganisms, i.e., at O_2_ concentrations of 1–20 μmol L^-1^ or higher (Kalvelage et al., 2011; Dalsgaard et al., 2014). Aggregates were produced in the laboratory using the ubiquitous, bloom-forming, and nitrate-storing diatom *Skeletonema marinoi*. Sinking diatom aggregates were individually studied at different ambient O_2_ levels to reveal the microscale O_2_ distribution inside the aggregates with microsensors and their inorganic nitrogen turnover with isotopically labeled nitrate.

## Materials and Methods

### *Skeletonema* Aggregates

Diatom aggregates were produced in the laboratory using cultured *Skeletonema marinoi* (CCMP1332, NCMA), natural seawater, and a plankton wheel. *S. marinoi* was cultured in F/2 medium plus silicate (Guillard and Ryther, 1962) prepared with filtered (0.45 μm) and autoclaved coastal seawater from the Baltic Sea (Kerteminde, Denmark) adjusted from 15 psu up to 30 psu with NaCl. The cultivation temperature was 14°C and the light:dark cycle was 10:14 h. For aggregate production, 50 mL of stationary-phase *S. marinoi* culture was mixed with 550 mL unfiltered coastal seawater from the same site and filled bubble-free into glass bottles. These aggregate production bottles were fixed to a plankton wheel (diameter: 60 cm) and continuously rotated to keep the diatom cells in suspension and to make them collide with each other and with microbes and small particles suspended in the seawater and thereby form aggregates. Spherical aggregates of 1 mm in diameter formed within 24 h and grew to larger, mostly ellipsoidal aggregates within 3 days. The rotation speed of the plankton wheel was repeatedly adjusted to make sure that the growing aggregates were continuously sinking rather than colliding with the wall of the aggregate production bottle, which would promote the compaction of the aggregates (Jackson, 2015). The seawater in each bottle was quantitatively replaced by fresh, aerated seawater on a daily basis to compensate for drops in O_2_ and NO_3_^-^ concentrations and to minimize bottle effects on the microbial community composition. The NO_3_^-^ concentration in the unamended seawater was in the range of 0.1–13.7 μmol L^-1^ (4.5 μmol L^-1^ on average). One day before experimentation with aggregates, the seawater was amended with 25 μmol L^-1^ ^14^NO_3_^-^ and the aggregate production bottles were wrapped in aluminum foil. This mimicked the shift in conditions that aggregates encounter when they sink out of the photic zone into the dark, nitrate-rich aphotic zone. Microsensor measurements and stable isotope experiments were made with 4–7 days old aggregates.

### Microsensor Profiling

Oxygen concentration profiles were measured through the center of sinking *Skeletonema* aggregates using microsensors in a net-jet flow system (Ploug and Jørgensen, 1999). Forty-two microprofiles were measured in 39 replicate aggregates exposed to 15, 40, 70, and/or 100% air saturation (AS) in coastal seawater adjusted to 25 μmol L^-1^ NO_3_^-^, 14°C, and darkness. One single aggregate was profiled at all four AS levels in random order, whereas the remaining aggregates were profiled at one AS level only. Oxygen microsensors were constructed and calibrated as described before (Revsbech, 1989), mounted on a motor-driven micromanipulator, and used for profiling at 100 μm increments.

### ^15^N-Stable-Isotope Incubations

Rates of NO_3_^-^ turnover by single *Skeletonema* aggregates were determined by incubation in ^15^NO_3_^-^-enriched seawater adjusted to different ambient O_2_ levels (*n* = 5–9). Control incubations without aggregates were run at each ambient O_2_ level (*n* = 3). All incubations were made at 14°C and in darkness. Gas-tight incubation vials (25-mL serum bottles) were filled with unfiltered seawater (see above) amended with 23 μmol L^-1^ ^15^NO_3_^-^ (98 atom% ^15^N; Sigma–Aldrich) and adjusted to AS levels of 0, 15, 40, 70, or 100% by flushing with the appropriate oxygen:helium mixture. Ellipsoidal aggregates with a short-axis length of at least 2.5 mm were selected from the aggregate production bottles and carefully picked with a glass tube. The length of the three axes of the aggregates was measured to 0.5 mm with a caliper. One aggregate was transferred into each of the incubation vials which were then sealed with a butyl rubber stopper. The incubation vials were wrapped in aluminum foil and rotated on the plankton wheel to keep the aggregates sinking. Oxygen measurements were made and samples for N analyses were withdrawn hourly for a total of 6 h. Oxygen concentration was measured with optode spots (SensorSpot, Pyroscience, Germany) fixed to the inside of the incubation vials and an optical O_2_ meter (FireStingO_2_, Pyroscience, Germany; **Supplementary Figure S1C**). For N analyses, a 2.5-mL water sample was taken with a syringe inserted through the stopper (**Supplementary Figure S1C**). A second syringe filled with 2.5 mL seawater (with known NO_3_^-^ and O_2_ concentrations) was inserted through the stopper and its content injected into the incubation vial while the first syringe was pulled up. To achieve efficient mixing, the reciprocal use of the two syringes was repeated twice. Care was taken not to destroy the aggregate during sampling. The 2.5-mL water sample was split into 1.5 mL for N_2_ and N_2_O analyses and 1 mL for dissolved inorganic nitrogen (DIN) analyses. The N_2_ and N_2_O sample was quickly injected into a helium-flushed and half-evacuated 3-mL exetainer (Labco, Wycombe, UK) that contained 50 μL ZnCl_2_ (50% w/v) to stop metabolic activities. The DIN sample was immediately frozen at –20°C.

Net turnover rates of O_2_ and N compounds (^15^N-labeled and total) were calculated from linear concentration changes during the incubation and corrected for the dilution due to repeated sampling (**Supplementary Figure S3**). Rates determined in control incubations without aggregates were subtracted from those determined in incubations with aggregates. Nitrite production indicated activity of *dissimilatory nitrate reduction to nitrite*, NH_4_^+^ production indicated activity of *dissimilatory nitrate reduction to ammonium* (*DNRA*), and N_2_O and N_2_ production indicated *denitrification* activity. For NO_3_^-^, NO_2_^-^, NH_4_^+^, and N_2_, the turnover rates were calculated for the ^15^N- and ^14^N-isotopes and the sum of them (i.e., total rates), whereas for N_2_O only total turnover rates could be calculated. Specifically, the NO_3_^-^_total_ and ^15^NO_3_^-^ turnover rates were directly measured and the ^14^NO_3_^-^ turnover rate was obtained by subtraction. Production rates of N_2total_, ^15^N-N_2_, and ^14^N-N_2_ were calculated from the directly measured ^29^N_2_ and ^30^N_2_ production rates assuming the principles of random isotope pairing in the absence of anammox in the freshly produced aggregates (Nielsen, 1992). Total NH_4_^+^ production rates were calculated as the sum of the production rates of the heavy isotopes (measured directly) and the light isotopes (inferred) assuming that these two rates had the same ratio as the ^15^N-N_2_ and ^14^N-N_2_ production ratio. Total NO_2_^-^ production rates were calculated assuming that ^14^NO_2_^-^ and ^15^NO_2_^-^ production rates had the same ratio as the ^14^NO_3_^-^ and ^15^NO_3_^-^ concentrations in the seawater.

### Intracellular Nitrate Analysis

To determine the initial content of intracellular nitrate (ICNO_3_), 20 aggregates from three aggregate production bottles were analyzed. Aggregates were sized as described above, transferred into pre-weighed sample tubes, and immediately frozen in liquid nitrogen to stop all metabolic activities. The aggregate and the adhering water were weighed in the sample tube and stored at –20°C. For ICNO_3_ extraction, the aggregate samples were exposed to three freeze-thaw cycles (Heisterkamp et al., 2012). Nitrate concentrations in the aggregate and seawater samples were measured as described below. The ICNO_3_ content of the aggregate was calculated from the NO_3_^-^ concentrations in the aggregate sample and in the seawater. Per-diatom-cell ICNO_3_ concentrations were obtained from cell counts in aggregates and the average cell volume of *S. marinoi* (Kamp et al., 2011).

### Nitrogen Analyses

Nitrate and nitrite were analyzed on an NO_x_ analyzer (CLD 66s, Eco Physics) using the VCl_3_ and NaI reduction assay, respectively (Braman and Hendrix, 1989; Yang et al., 1997). Ammonium was analyzed with the salicylate method (Bower and Holm-Hansen, 1980). Isotopically labeled dinitrogen (^15^N-N_2_) was analyzed in the headspace of exetainer samples on a gas chromatography-isotopic ratio mass spectrometer (GC-IRMS; Thermo Delta V Plus, Thermo Scientific) with the excess above natural abundance calculated according to Nielsen (1992). Nitrous oxide was analyzed in the same exetainers on a gas chromatograph (GC 7890, Agilent Technologies). Both the prior sampling of the headspace for ^15^N-N_2_ analysis and the amount of nitrous oxide dissolved in the seawater were accounted for in the calculation of the total amount of nitrous oxide in the sample. ^15^N-labeled NO_3_^-^, NO_2_^-^, and NH_4_^+^ were analyzed with the cadmium/sulfamic acid, sulfamic acid, and hypobromite assay, respectively, followed by ^15^N-N_2_ analysis on the GC-IRMS (Warembourg, 1993; McIlvin and Altabet, 2005; Füssel et al., 2012).

## Results

### Characteristics of the Aggregates

Diatom aggregates were produced on a plankton wheel from axenic cultures of *S. marinoi* and the natural microbial community of coastal seawater (see sketch in **Supplementary Figure S1A**). Aggregates formed within 1–3 days, were ellipsoidal, had a smooth surface, and were dark brown (**Supplementary Figure S1B**). Aggregates used in ^15^NO_3_^-^ incubations had average dimensions of 6.1 mm × 4.0 mm × 3.0 mm, resulting in an average volume of 42 ± 24 mm^3^ (±SD, *n* = 36 aggregates). The average sinking velocity of *Skeletonema* aggregates of this average volume was 1055 ± 91 m d^-1^ (±SD, *n* = 10 aggregates) as determined in a sedimentation column.

**FIGURE 1:**
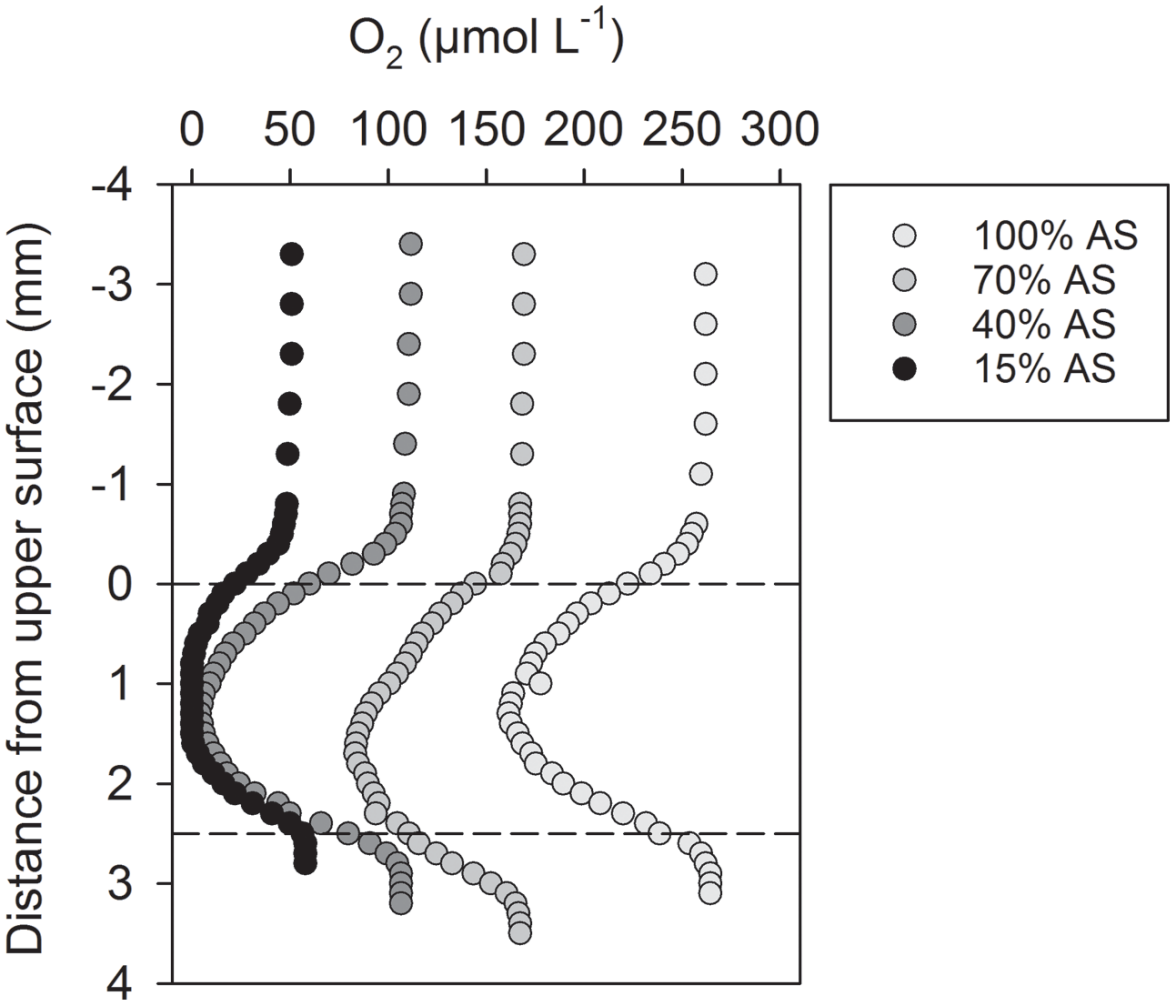
**Oxygen microsensor profiles through the center of a representative *Skeletonema* aggregate exposed to different air saturation (AS) levels in a net-jet flow system.** The sinking aggregate was incubated in coastal seawater adjusted to 25 μmol L^-1^ NO_3_^-^, at 14°C, and in darkness. Dashed lines indicate the upper and lower surface of the aggregate. Microsensor data measured in replicate aggregates are shown in **Supplementary Figure S2**.

The microscale O_2_ distribution inside dark-incubated, sinking *Skeletonema* aggregates was determined with O_2_ microsensors in a net-jet flow system (Ploug and Jørgensen, 1999). The microsensor profiles through the center of replicate aggregates revealed internal O_2_ concentrations that were always lower than the ambient O_2_ level (**Figure 1**, **Supplementary Figure S2**). Anoxic conditions in the center of a representative aggregate prevailed at ambient O_2_ concentrations that corresponded to 15 and 40% AS in the seawater (30 psu, 14°C; **Figure 1**). A large number of O_2_ microprofiles measured at 70 and 100% AS revealed a correlation between internal and ambient O_2_ concentration, according to which anoxia inside the diatom aggregates studied here was expected at 40% AS (i.e., ∼100 μmol O_2_ L^-1^) and below (**Supplementary Figure S2**).

### Nitrate Turnover at Different Ambient Oxygen Levels

The potential for dissimilatory nitrate reduction (DNR) activity of *Skeletonema* aggregates was tested at five ambient O_2_ levels by tracing the fate of ^15^NO_3_^-^ in short-term incubations (**Supplementary Figures S1A,C**). Thirty-six handpicked *Skeletonema* aggregates with a short-axis length of >2.5 mm were individually incubated in sealed glass vials that were mounted on a plankton wheel to simulate the sinking of the aggregates out of the photic zone (light, oxygen-rich, nitrate-poor) into the aphotic zone (dark, oxygen-poor, nitrate-rich). Concentration changes of NO_3_^-^, NO_2_^-^, NH_4_^+^, N_2_O, and N_2_, and the isotopic composition of these species were monitored for 6 h at 14°C. Stable O_2_ concentrations (**Supplementary Figure S3A**) were maintained by adjusting the O_2_ concentration in the refilled seawater at every sampling occasion according to measurements with an O_2_ optode fixed to the inside of the vials (**Supplementary Figure S1C**).

**FIGURE 2:**
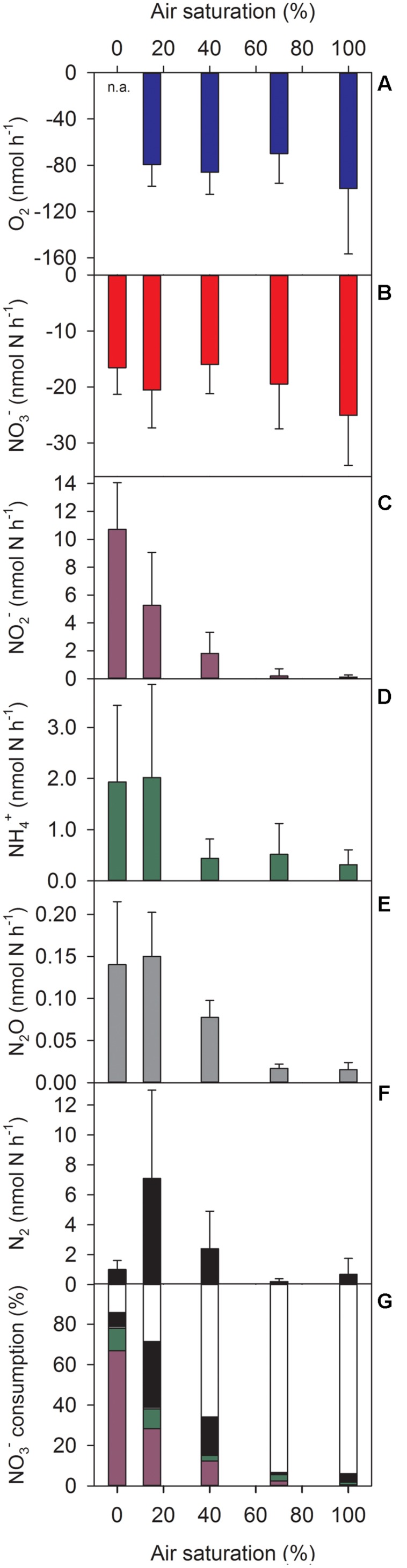
**Turnover rates of **(A–F)** O_2_, NO_3_^-^, NO_2_^-^, NH_4_^+^, N_2_O, and N_2_ and **(G)** mass balance of NO_3_^-^ consumption in sinking *Skeletonema* aggregates at different ambient air-saturation levels.** **(A–F)** Positive and negative rates correspond to production and consumption, respectively. **(G)** Color code of stacked bars refers to colors used in **(B–F)**; white indicates NO_3_^-^ consumption due to intracellular storage and/or assimilation. Means (+SD) of 5–9 individually incubated aggregates are shown. n.a., not applicable.

Oxygen (when present) and NO_3_^-^ were consumed at all ambient O_2_ levels tested (**Figures 2A,B**) with the consumption rates not being significantly affected by the ambient O_2_ level (**Supplementary Table S1**). In contrast, DNR activities of the aggregates, measured as net production rates of NO_2_^-^, NH_4_^+^, N_2_O, and N_2_, were significantly affected by the ambient O_2_ level (**Figures 2C–F**, **Supplementary Table S1**). High production rates were only measured at ambient O_2_ levels corresponding to 0–40% AS. At 70–100% AS, the production rates were generally low and sometimes not significantly different from zero (**Supplementary Table S2**).

A mass balance of NO_3_^-^ consumption (freely dissolved ^14^NO_3_^-^ and ^15^NO_3_^-^) was made based on the production rates of NO_2_^-^, NH_4_^+^, N_2_O, and N_2_. On average, the combined production of these N compounds amounted to 86, 72, and 34% of NO_3_^-^ consumption at 0, 15, and 40% AS, respectively (**Figure 2G**). Nitrite and N_2_ were quantitatively the most important products of NO_3_^-^ consumption, with a clear NO_2_^-^ dominance at 0% AS and about equal shares at 15 and 40% AS. At 70–100% AS, however, only a minor fraction of the NO_3_^-^ consumed in the incubations was retrieved as NO_2_^-^, NH_4_^+^, N_2_O, or N_2_ (**Figure 2G**). Thus, DNR activities explained the biggest proportion of NO_3_^-^ consumption (up to 14.7 nmol aggregate^-1^ h^-1^) at low ambient O_2_ levels, whereas other processes (e.g., NO_3_^-^ storage and assimilation) were relatively more important at high ambient O_2_ levels.

Nitrification activity of *Skeletonema* aggregates was assessed from temporal changes in the ^14^NO_3_^-^/^15^NO_3_^-^ concentration ratio. No consistent trend of increasing ^14^NO_3_^-^/^15^NO_3_^-^ concentration ratios (which would indicate ^14^NO_3_^-^ release from the aggregates due to nitrification activity) was observed (**Supplementary Figure S4**). Instead, the residuals of a linear regression of the ^14^NO_3_^-^/^15^NO_3_^-^ concentration ratios at *t*_0h_ vs. *t*_6h_ were normally distributed, indicating analytical noise. Nitrification rates as low as 0.1 nmol NO_3_^-^ h^-1^ per aggregate would be detected with this approach. It can thus not be ruled out that individual aggregates exhibited nitrification activity lower than that rate.

### Diffusion Limitation of Oxygen and Nitrate Consumption

The ^15^NO_3_^-^ incubations at different O_2_ levels covered a considerable size range of aggregates (i.e., 16–132 mm^3^). Volumetric rates of O_2_ and NO_3_^-^ consumption calculated from individual aggregate volumes and per-aggregate rates (i.e., rates in aggregate incubations minus rates in control incubations; **Figures 2A,B**) were significantly, negatively correlated with the equivalent spherical radius of the aggregates (**Supplementary Figures S5A,B**). The corresponding power functions had exponents of –2.3 and –2.6 for O_2_ and NO_3_^-^, respectively, in the ellipsoidal aggregates and were thus close to the theoretical exponent of –2 for diffusion-limited solute uptake by spherical aggregates (Jørgensen, 1977; Schramm et al., 1999). Hence, solute transport inside the sinking diatom aggregates occurred through diffusion rather than advection.

### Internal Nitrate Availability

**FIGURE 3:**
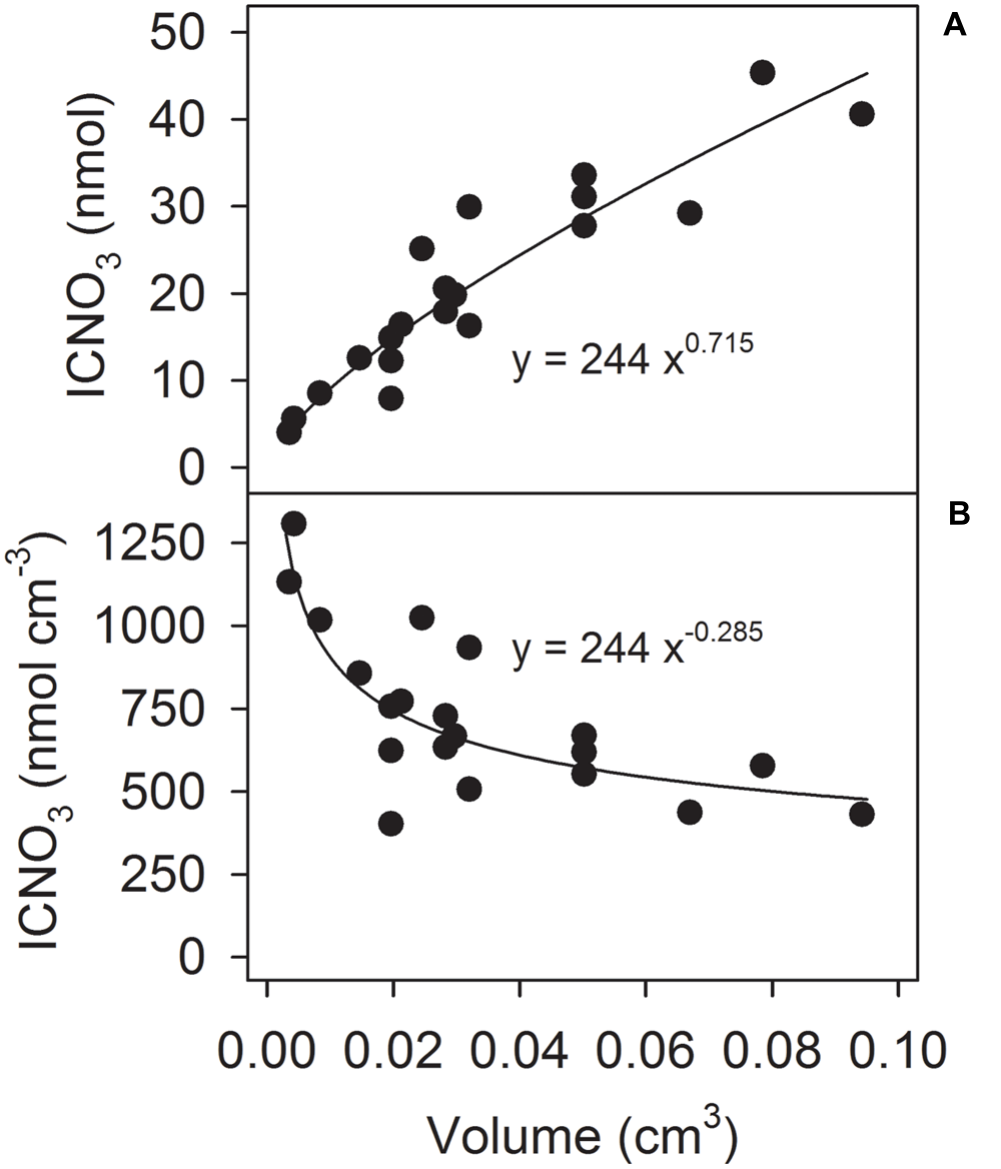
**Correlation between aggregate volume and **(A)** ICNO_3_ content and **(B)** ICNO_3_ concentration (per aggregate volume) in 20 freshly produced *Skeletonema* aggregates**.

Aside from the added ^15^NO_3_^-^, the incubations contained low amounts of ^14^NO_3_^-^ freely dissolved in the seawater. Strikingly though, the ^14^N-N_2_/^15^N-N_2_ concentration ratios were much higher throughout the incubations than the ^14^NO_3_^-^/^15^NO_3_^-^ concentration ratio (**Supplementary Figure S6**). The excess ^14^N-N_2_ production deduced from this observation hints to the presence of an additional ^14^NO_3_^-^ source inside the aggregates. The time course of the ^14^N-N_2_/^15^N-N_2_ concentration ratios further suggests an early phase of consumption of ^14^NO_3_^-^ that is already present inside the aggregate at the beginning of the incubation and a later phase of consumption of ^14^NO_3_^-^ that is generated or released into the aggregate during the incubation (**Supplementary Figure S6C**). Since *S. marinoi* is known to store NO_3_^-^ in their cells (Kamp et al., 2011), intracellular nitrate (ICNO_3_) was analyzed in 20 freshly produced *Skeletonema* aggregates. The total ICNO_3_ contents amounted to 4–45 nmol N aggregate^-1^, correlated well with aggregate volume (**Figure 3A**), and corresponded to volumetric ICNO_3_ concentrations of 0.4–1.3 μmol N cm^-3^, with higher concentrations in smaller aggregates (**Figure 3B**). The corresponding per-diatom-cell concentrations were 39–59 mmol ICNO_3_ L^-1^. These ICNO_3_ concentrations were measured 4–7 days after the washed diatom cells had been transferred from culture medium (with initially 1 mmol NO_3_^-^ L^-1^) into natural seawater (with 0.1–13.7 μmol NO_3_^-^ L^-1^) and thus likely reflect ICNO_3_ levels that *S. marinoi* maintains at low ambient NO_3_^-^ concentrations. Furthermore, the ^15^N-stable-isotope enrichment experiments were not compromised by the ICNO_3_ present at the onset of the incubations. Even the maximum total ICNO_3_ content of 56 nmol estimated for one of the aggregates used for ^15^NO_3_^-^ incubation decreased the labeling percentage of NO_3_^-^ in the incubation bottle only from 87 to 80% and thus still allowed high-quality isotopic N_2_ analysis.

**FIGURE 4:**
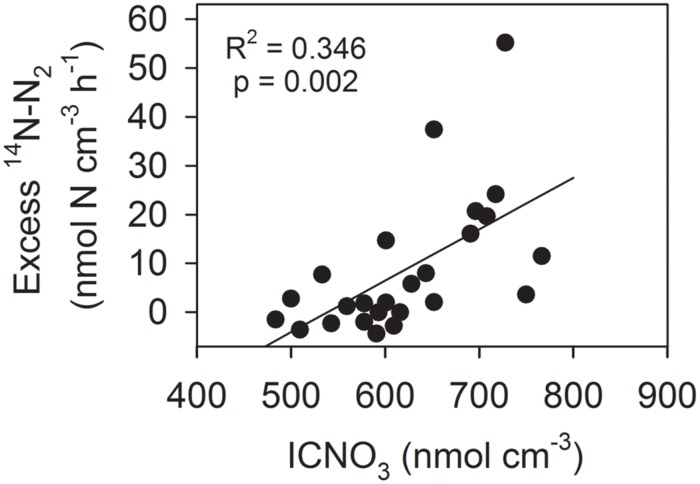
**Correlation between estimated initial ICNO_3_ concentration and excess ^14^N-N_2_ production rate (i.e., the difference between measured and predicted ^14^N-N_2_ production) in 25 *Skeletonema* aggregates incubated with ^15^NO_3_^-^ at 0–40% air saturation**.

Using the regression equation presented in **Figure 3B**, the initial ICNO_3_ concentration of each ^15^NO_3_^-^-incubated aggregate was estimated. The excess ^14^N-N_2_ production (i.e., the difference between the measured ^14^N-N_2_ production rate and the ^14^N-N_2_ production rate predicted from the initial ^14^NO_3_^-^/^15^NO_3_^-^ concentration ratio) was significantly, positively correlated with the initial estimated ICNO_3_ concentration of the aggregates (**Figure 4**). Consumption rates of ICNO_3_ estimated from the excess ^14^N-N_2_ production rates were very low with 0.23–0.27 nmol N aggregate^-1^ h^-1^ at 0–40% AS and only 0.06 nmol N aggregate^-1^ h^-1^ at 70–100% AS. The amount of ICNO_3_ consumed during the 6-h incubation at 0–40% AS corresponded to only 6.2–10.8% of the estimated initial ICNO_3_ contents. It cannot be ruled out that ICNO_3_ was also actively stored following the step-up of ambient NO_3_^-^ concentration at the beginning of the incubations, especially at the high ambient O_2_ levels, where it would have contributed to the large fraction of NO_3_^-^ consumption not accounted for by DNR activities (**Figures 2B,G**).

## Discussion

### Diatom Aggregates Host Anaerobic Nitrogen-Cycle Pathways

The aggregates produced from the ubiquitous diatom *S. marinoi* showed high DNR rates at ambient O_2_ levels that were 1–2 orders of magnitude higher than inhibitory levels for DNR by free-living microorganisms (Kalvelage et al., 2011; Dalsgaard et al., 2014). Significant DNR rates were measured at ambient O_2_ concentrations ≤ 100 μmol L^-1^ (i.e., 40% AS), at and below which anoxic conditions prevailed in the center of the aggregates, as confirmed with microsensor measurements (**Figure 1**, **Supplementary Figure S2**). Community respiration inside the aggregates thus exceeded the supply of O_2_ from the surrounding water (Jørgensen, 1977; Ploug et al., 1997; Ploug and Bergkvist, 2015). As a consequence, facultative anaerobic microorganisms (prokaryotes and/or eukaryotes) inside the aggregates must have switched from aerobic respiration to DNR processes. Products and intermediates of these DNR processes were released into the surrounding water. Natural diatom aggregates with similar characteristics thus have the potential to contribute to fixed-nitrogen loss (as N_2_), directly through denitrification and indirectly by supplying NO_2_^-^ and NH_4_^+^ for anammox, and to greenhouse gas production (as N_2_O) in the water column of the ocean, even at relatively high ambient O_2_ levels.

Dissimilatory nitrate reduction was not the only significant NO_3_^-^ sink in *Skeletonema* aggregates. The relative share of DNR in NO_3_^-^ consumption decreased gradually from ∼85% to ∼5% with the ambient O_2_ level increasing from 0 to 100% AS (**Figure 2G**). The unaccounted fraction of NO_3_^-^ consumption was probably due to cellular uptake and storage and/or assimilation of NO_3_^-^. Many diatoms, including *S. marinoi*, store NO_3_^-^ intracellularly at concentrations by far exceeding ambient levels, mostly for assimilatory or dissimilatory use (Lomas and Glibert, 2000; Kamp et al., 2011, 2015). Intracellular nitrate (ICNO_3_) analysis in our *Skeletonema* aggregates revealed total contents of 4–45 nmol ICNO_3_ per aggregate, whereas the NO_3_^-^ consumption unaccounted for by DNR amounted to 14–141 nmol NO_3_^-^ at the end of the 6-h incubations at 0–100% AS. Thus, a substantial fraction of non-dissimilatory NO_3_^-^ consumption might be due to ICNO_3_ storage by *S. marinoi*, and the rest must be ascribed to NO_3_^-^ assimilation by the microbial community of the aggregate.

Nitrite was quantitatively the most important product of DNR released from *Skeletonema* aggregates, followed by N_2_, NH_4_^+^, and N_2_O. In natural marine snow, NO_2_^-^ and NH_4_^+^ concentrations are significantly higher than in the ambient water (Shanks and Trent, 1979; Kaltenböck and Herndl, 1992), which in the light of our results might be due to aggregate-associated DNR. In contrast, NO_3_^-^ concentrations in natural marine snow are not consistently higher than ambient concentrations and sometimes even lower (Shanks and Trent, 1979; Alldredge and Gotschalk, 1989; Kaltenböck and Herndl, 1992). Lower NO_3_^-^ concentrations in aggregates are consistent with DNR activities, whereas higher NO_3_^-^ concentrations may result from nitrification activity, but also from NO_3_^-^ measurements that include the intracellular NO_3_^-^ stores of diatoms because of cell leakage during sample preservation or processing.

Nitrite rather than N_2_ was the main product of DNR also at 0% AS. In OMZs, NO_2_^-^ accumulation due to DNR is frequently observed at high NO_3_^-^ concentrations (Lam et al., 2011; Kalvelage et al., 2013; Ganesh et al., 2015). The ambient NO_3_^-^ concentration in our incubations of ∼25 μmol L^-1^ corresponds to the high NO_3_^-^ concentrations typically encountered in OMZs (Anderson et al., 1982; Ganesh et al., 2015). However, the inverse relationship between the volumetric consumption rate of ambient NO_3_^-^ and the aggregate radius suggested diffusional NO_3_^-^ limitation inside the aggregate (Schramm et al., 1999). Therefore, N_2_ rather than NO_2_^-^ was the expected end product of aggregate-associated DNR, as is the case in marine sediments with diffusional NO_3_^-^ limitation in the denitrification layer (Devol, 2015). It may be speculated, however, that the ICNO_3_ pool of the densely packed diatoms in the aggregates, if released into the aggregate due to microbial attack and cell lysis, will abolish this NO_3_^-^ limitation and promote NO_3_^-^ reduction to NO_2_^-^ only.

The negligible aggregate-associated nitrification activity is surprising because NH_4_^+^ was not quantitatively released into the surrounding water. Only at low ambient O_2_ levels (0 and 15% AS), about half of the aggregates released significant amounts of NH_4_^+^. Theoretical NH_4_^+^ production rates estimated from the measured rates of O_2_ consumption and DNR (and assuming degradation of ‘Redfieldian’ organic matter) amount to ∼10–15 nmol NH_4_^+^ h^-1^, which should lead to a substantial NH_4_^+^ release in the absence of nitrification. An alternative sink for NH_4_^+^ inside the aggregates may have been NH_4_^+^ assimilation by diatoms and bacteria. If nitrification activity was mainly located close to the oxic-anoxic interface inside the aggregate, then part of the newly produced ^14^NO_3_^-^ would have diffused into the anoxic core and contributed to the production of ^14^N-N_2_. The excess ^14^N-N_2_ production, however, was as low as 0.23–0.27 nmol N h^-1^ and was significantly correlated with the ICNO_3_ concentration of the aggregates. Additionally, there is no evidence for NH_4_^+^ consumption in the subsurface layer or for NH_4_^+^ limitation in the oxic surface layer of the aggregates provided by microsensor measurements. Low or negligible nitrification rates were also measured in natural cyanobacterial and laboratory-made diatom aggregates (Klawonn et al., 2015; Ploug and Bergkvist, 2015). The generally low growth rates of nitrifying microorganisms may delay the colonization of short-lived sinking aggregates. Moreover, nitrifiers might be outcompeted by heterotrophic bacteria inside the organic-carbon-rich aggregates (Strauss and Lamberti, 2000).

### Conceptual Model of Nitrogen Cycling in Diatom Aggregates

**FIGURE 5:**
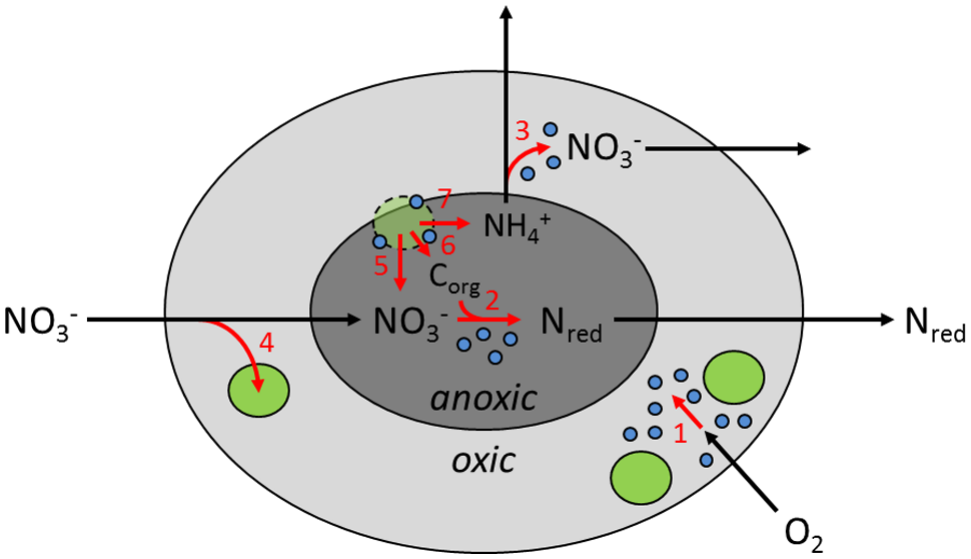
**Conceptual model of inorganic nitrogen metabolism in sinking diatom aggregates in the marine pelagial, highlighting the spatial organization of microenvironments, microbes, and metabolic activities (not to scale).** Light and dark gray areas represent oxic and anoxic compartments inside the aggregate, respectively. Green and blue circles represent diatom and bacterial cells, respectively. Lysing diatom cells have dashed contours. N_red_ denotes the sum of any product of dissimilatory nitrate reduction. Black arrows indicate transport of solutes, whereas red arrows indicate microbial turnover of solutes: (1) aerobic respiration, (2) dissimilatory nitrate reduction, (3) nitrification, (4) nitrate storage and/or assimilation by diatoms, (5) nitrate leakage by lysing diatoms, (6) organic carbon (C_org_) leakage by diatoms, and (7) DNRA activity by diatoms.

Marine snow is highly enriched in microbial biomass, exceeding ambient densities by three orders of magnitude (Ploug et al., 1999; Grossart et al., 2003; Thiele et al., 2015). Nevertheless, at total aggregate volumes of only a few cm^3^ per m^3^ water, the aggregate-associated microbial biomass makes up only little of the total microbial biomass (Thiele et al., 2015). It is thus much more the spatial structure of this concentrated biomass by which sinking aggregates leave their mark on oceanic water-column biogeochemistry. Physical, chemical, and biological gradients inside the aggregates persist within a relatively homogeneous macroenvironment. This microscale gradient system allows metabolic activities that otherwise would not occur in the water column, obviously also anaerobic nitrogen cycling at otherwise inhibitory ambient O_2_ levels. The diatom aggregates used in this study had sufficient size and respiration rates to sustain an anoxic center even at intermediate O_2_ levels in the ambient water (**Figure 5**). In spherical or ellipsoidal aggregates in which diffusional solute exchange dominates, a near-concentric arrangement of aggregate surface, oxic-anoxic interface, and NO_3_^-^ penetration depth may establish (Klawonn et al., 2015). We propose that DNR activity proceeds just below the oxic-anoxic interface inside diatom aggregates.

Our experiments with sinking diatom aggregates also revealed that DNR activities can be uncoupled from ambient NO_3_^-^ supply due to the ICNO_3_ stores of the aggregate-associated diatoms. Based on the estimates obtained in these experiments, the ICNO_3_ stores of lysing diatom cells may sustain DNR activities of the microbial community of the aggregates for 54–96 h, thus for most of the sinking period of natural aggregates even through deep parts of the ocean. In contrast, DNRA activity of living diatom cells exposed to anoxic conditions inside the aggregates may only last for a few hours until the ICNO_3_ stores are used up (Kamp et al., 2011). Depending on the ICNO_3_ consumption rate, sinking velocity, and water depth, diatom aggregates will export a so far unquantified amount of NO_3_^-^ to deep water layers or even the sea floor. Fast-sinking aggregates may deliver ICNO_3_ all the way to the seafloor where microbial degradation of the aggregates can be expected to create anoxic microsites in otherwise oxic sediments and liberate NO_3_^-^ as well as labile organic carbon from lysing diatom cells (Glud, 2008). Settled aggregates are therefore thought to stimulate benthic denitrification and other DNR processes (Lehto et al., 2014).

We hypothesize that diatoms contribute to DNR activities in marine snow, both directly and indirectly (**Figure 5**): (1) Diatoms near the surface of the aggregate take up and store NO_3_^-^ under oxic conditions in photic water layers and thereby contribute to overall NO_3_^-^ consumption by the aggregate. (2) Under dark conditions, diatoms near the oxic-anoxic interface within the aggregate use their ICNO_3_ stores for DNRA and thereby directly contribute to DNR by the aggregate. (3) In the anoxic center of the aggregates, diatom cells lyse and release ICNO_3_ into the aggregate (and potentially also labile organic matter), which fuels DNR by the microbial community of the aggregate. Diatom aggregates thus release NH_4_^+^ through both the mineralization of organic matter (Ploug and Bergkvist, 2015) and the DNRA activity of the diatoms (Kamp et al., 2011, 2013). In OMZs, diatom aggregates may therefore play a key role by supplying NH_4_^+^ to the anammox process, a still debated issue (Dalsgaard et al., 2012; Kalvelage et al., 2013; Babbin et al., 2014).

Microbial nitrogen cycling associated with marine snow apparently differs between diatom and cyanobacterial aggregates. Denitrification was not detected in ^15^N-stable-isotope incubations of deoxygenated cyanobacterial biomass enriched from a bloom in the Baltic Sea during which aggregates were observed (Hietanen et al., 2002). Negligible denitrification activity was measured in ^15^N-stable-isotope incubations of intact cyanobacterial aggregates (*Nodularia* sp.) collected in the Baltic Sea and incubated under oxic conditions, despite the presence of denitrification genes (Tuomainen et al., 2003) and anoxic centers in the aggregates as measured with microsensors (Klawonn et al., 2015). In contrast, DNRA rates were similarly high in cyanobacterial aggregates (Klawonn et al., 2015) as in the *Skeletonema* aggregates. The reasons for the insignificance of denitrification, but not DNRA, in cyanobacterial aggregates are currently not known.

### Ecological Implications of Nitrogen Cycling in Diatom Aggregates

The diatom aggregates studied here had sinking velocities in the upper range of what has been reported for other marine aggregates (Diercks and Asper, 1997; Ploug et al., 2008; Villareal et al., 2011; Turner, 2015). Diatom aggregates produced in the laboratory may reach sinking velocities > 800 m d^-1^ (Ziervogel and Forster, 2005; Iversen and Ploug, 2013), whereas natural diatom aggregates have sinking velocities of 50–200 m d^-1^ (Alldredge and Gotschalk, 1989; Ploug et al., 2008) or sometimes 500 m d^-1^ (Iversen and Ploug, 2010). High sinking velocities result from a dense packing of organic matter, microbial biomass, and ballast materials (Ploug et al., 2008). The ensuing high mineralization and respiration rates inside compact aggregates cause steep O_2_ concentration gradients, which makes anoxia and DNR activity more likely to occur inside the aggregate. In contrast, high sinking velocities decrease the thickness of the surrounding diffusive boundary layer at the upstream side of the aggregate and thereby potentially increase the O_2_ flux to the aggregate (Ploug, 2001; Ploug et al., 2008). As a consequence, the hypoxic or anoxic center of the aggregate can be shifted to the downstream side (Ploug et al., 1997) or may even disappear and render DNR activity unlikely. The question remains whether anoxia and DNR activity are also likely to occur in less compact and slower-sinking aggregates. Such “fluffy” aggregates probably possess lower respiration rates (anoxia less likely) and thicker diffusive boundary layers (anoxia more likely), but may also be affected by advective porewater flow (anoxia less likely). Importantly, the residence time of slow-sinking aggregates in deep, hypoxic or anoxic water layers is prolonged, which will increase time-integrated DNR rates. Studies on less compact and/or smaller aggregates concluded that internal anoxia should be common at <20–25 μmol O_2_ L^-1^ in the ambient water (Ploug, 2001; Klawonn et al., 2015).

The quantitative data on the relatively large and compact diatom aggregates used in this study cannot be directly extrapolated to any marine environment where smaller and less compact aggregates are exposed to high O_2_ levels. However, in the productive coastal upwelling regions adjacent to OMZs, the export flux of particulate organic carbon is high (Muller-Karger et al., 2001; Chavez et al., 2011), dominated by diatom aggregates (Kiørboe et al., 1998; Wright et al., 2012), and ambient O_2_ levels are low. Approximately 0.08–0.11 g C m^-2^ d^-1^ reaches the anoxic water body within the Cariaco Basin (Muller-Karger et al., 2001). In the Peruvian upwelling system, 0.08–1.26 g C m^-2^ d^-1^ reaches the upper boundary of the Eastern Tropical South Pacific OMZ (Kalvelage et al., 2013). These carbon export fluxes would correspond to densities of 1.5–2.0 and 1.5–24 *Skeletonema* aggregates m^-3^, respectively (on the basis of average-sized aggregates with an organic carbon content of 50 μg (estimated from Iversen and Ploug, 2013, Figure 3A and a sinking velocity of 1055 m d^-1^). Combining these ranges of aggregate densities, N_2_ production mediated by anoxic diatom aggregates would be equivalent to 0.13–2.04 nmol N_2_ L^-1^ d^-1^, which is at the lower end of fixed-nitrogen loss rates of 0–90 nmol N_2_ L^-1^ d^-1^ reported for OMZs (Lam and Kuypers, 2011). High rates are, however, typically restricted to the upper 50–100 m of the anoxic OMZ core, and thus the aggregate-associated rates would more likely constitute a substantial contribution deeper in the OMZs where N_2_ production is often not detectable in experimental incubations, which would typically not include the aggregates (Dalsgaard et al., 2012; Kalvelage et al., 2013).

During seasonal pulses of pelagic primary production like phytoplankton blooms and the ensuing mass sinking of aggregates (Smetacek, 1985; Thornton, 2002), aggregate densities in coastal regions can be as high as 0.1–15 aggregates L^-1^ or >1000 times higher than the values calculated above (Knauer et al., 1982; Lampitt et al., 2001; Grossart et al., 2003). Additionally, natural diatom aggregates in coastal regions cover a size range of 0.16–523 mm^3^ (Grossart et al., 2003), meaning that large specimens can be >10 times bigger than an average-sized diatom aggregate of this study. At such high *in situ* densities and sizes, aggregate-associated DNR could in fact be even more significant than calculated above. However, experimentation with natural aggregates and modeling efforts accounting for variations in size and compactness of aggregates are needed to arrive at better estimates of the contribution of marine snow to DNR activities in the marine pelagic.

The quantitative importance of sinking diatom aggregates for pelagic DNR can also be assessed with respect to the total ocean volume in which DNR can potentially take place due to the presence of aggregates. Globally, the ocean volume with O_2_ concentrations ≤100 μmol L^-1^ (at which aggregate-associated DNR occurred in this study) is >90 times larger than the volume with ≤5 μmol L^-1^ (at which DNR by free-living microbes is possible; Karstensen et al., 2008; Kalvelage et al., 2011; Dalsgaard et al., 2014). Even for the threshold concentration of 20–25 μmol O_2_ L^-1^ proposed to support anoxia in other studies (Ploug, 2001; Klawonn et al., 2015), this factor would still be >20. Thus, integrated over the oceans, aggregate-associated DNR is probably more important at the boundaries of OMZs than within their core. Even at relatively low bulk rates in these voluminous areas, aggregate-associated nitrogen turnover and fixed-nitrogen loss, in particular, could contribute substantially to oceanic nitrogen budgets.

## Conclusion

The findings of this study provide a new conceptual basis for aggregate-scale to ecosystem-scale modeling and understanding of nitrogen cycling, especially in low-oxygen environments. Extending the existing models of pelagic nitrogen cycling by aggregate-related processes is essential for both the notorious and the emerging ecosystems with high aggregate densities, such as eutrophic coastal regions (Müller-Niklas et al., 1994), oceanic OMZs (Wright et al., 2012), Arctic waters impacted by sinking aggregates of sea-ice algae (Boetius et al., 2013), and even the deep sea (Bochdansky et al., 2010). Since all of the aforementioned marine ecosystems are characterized by relatively high NO_3_^-^ concentrations, the contribution of sinking aggregates to fixed-nitrogen loss needs to be better constrained.

## Author Contributions

PS, RG, and BT designed the study. PS carried out the ^15^N-labeling experiments. AK made the intracellular nitrate measurements. All authors interpreted the data. PS wrote the manuscript with input from all co-authors.

## Conflict of Interest Statement

The authors declare that the research was conducted in the absence of any commercial or financial relationships that could be construed as a potential conflict of interest.
