# Supplementary material for: Anaerobic Nitrogen Turnover by Sinking Diatom Aggregates at Varying Ambient Oxygen Levels
Source: Front Microbiol. 2016 Feb 5;7:98. doi: 10.3389/fmicb.2016.00098 (PMC4742529; doi:10.3389/fmicb.2016.00098)
Supplement: Supplementary file 1 [file Presentation_1.PDF]

Supplementary Information

Anaerobic Nitrogen Turnover by Sinking Diatom Aggregates at Varying Ambient Oxygen Levels

Peter Stief [1,*], Anja Kamp[2], Bo Thamdrup[1], and Ronnie N. Glud[1]

[1]Department of Biology and Nordic Center for Earth Evolution,

University of Southern Denmark, Odense, Denmark

[2]AIAS, Aarhus Institute of Advanced Studies, Aarhus University, Aarhus, Denmark

*Department of Biology and Nordic Center for Earth Evolution,

University of Southern Denmark, Campusvej 55, 5230 Odense M, Denmark

Phone: +45 6550 7980

E-mail: peterstief@biology.sdu.dk

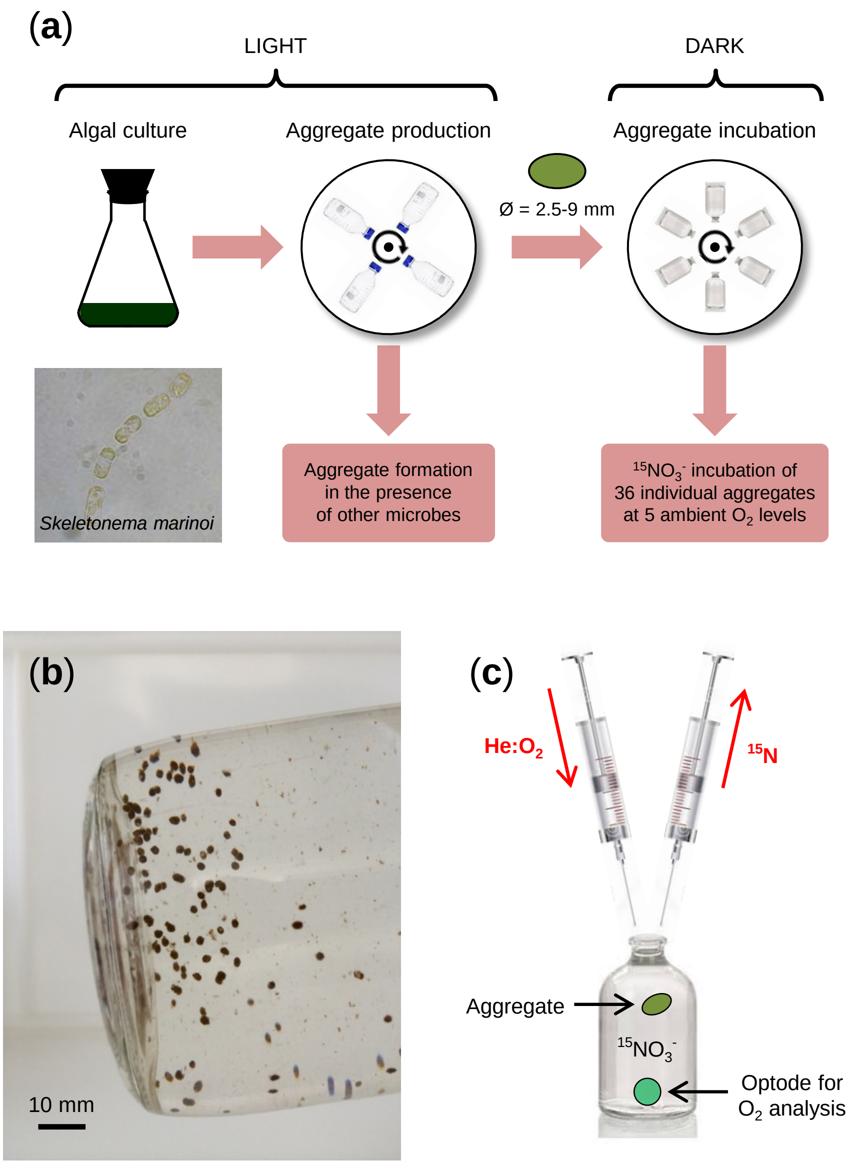

Figure S1: (**a**) Sketch of the production of diatom aggregates from axenic *Skeletonema marinoi* and the microbial community of coastal seawater on a plankton wheel and the subsequent incubation of *Skeletonema* aggregates with $^{15}NO_3^-$ at different ambient O_2 levels. (**b**) Picture of sinking *Skeletonema* aggregates in an aggregate production bottle. (**c**) Sketch of the $^{15}NO_3^-$-incubation experiment with an individual *Skeletonema* aggregate suspended in a glass bottle that is equipped with an optode spot for O_2 measurements; the sampling procedure with two reciprocally used syringes is shown.

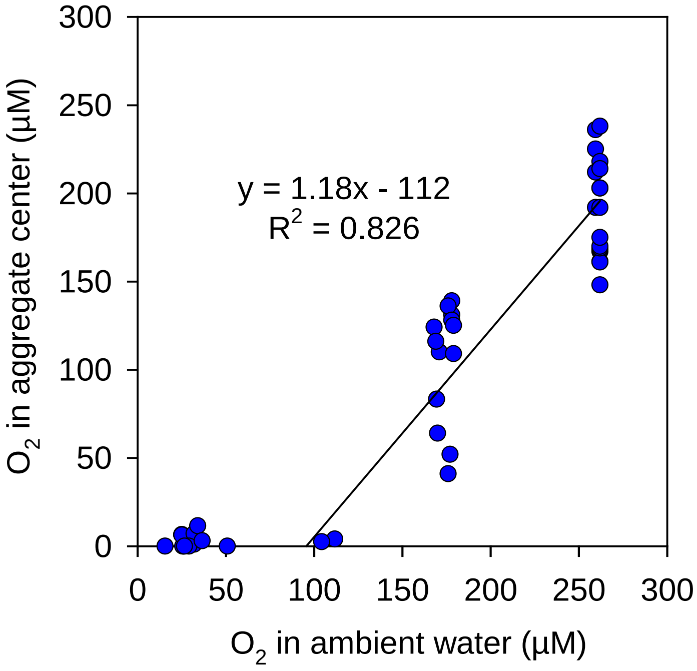

Figure S2: Minimum O_2 concentrations inside 39 similarly sized *Skeletonema* aggregates as measured with microsensors in a net-jet flow system. The minimum internal O_2 concentration is plotted against ambient O_2 concentration. A linear regression was calculated for the data points obtained at 40, 70, and 100% air saturation (i.e., 105, 183, and 262 µmol O_2 L^{-1}), which indicated that in the diatom aggregates studied here, internal anoxia is expected at 40% air saturation (i.e., 105 µmol O_2 L^{-1}) and below.

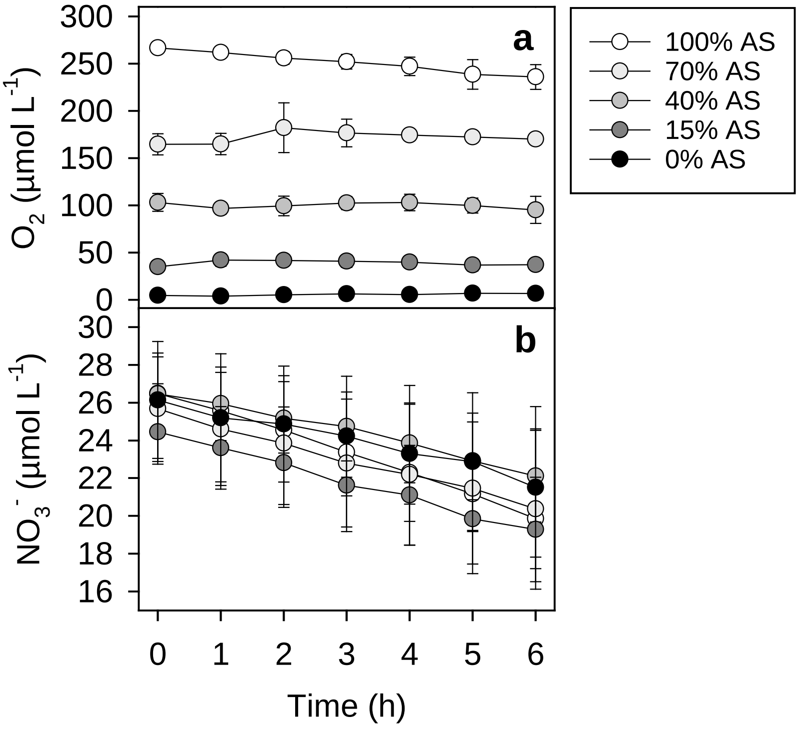

Figure S3: Concentration time series of (**a**) O_2 and (**b**) NO_3^- at different air saturation (AS) levels in the $^{15}NO_3^-$-incubation experiment. Means (±SD) of 5-9 replicates are shown. Note the stable O_2 concentrations and the linear decrease of NO_3^- concentrations over time.

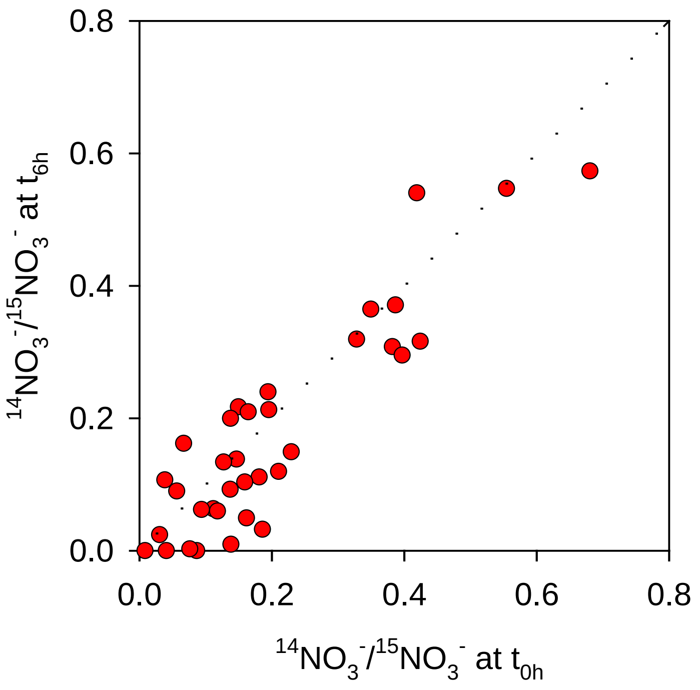

Figure S4: Assessment of nitrification activity by *Skeletonema* aggregates based on temporal changes of the $^{14}NO_3^-/^{15}NO_3^-$ concentration ratio at t_{0h} vs. t_{6h}. No consistent trend of increasing $^{14}NO_3^-/^{15}NO_3^-$ concentration ratios (which would indicate $^{14}NO_3^-$ release by the aggregates due to nitrification activity) was observed.

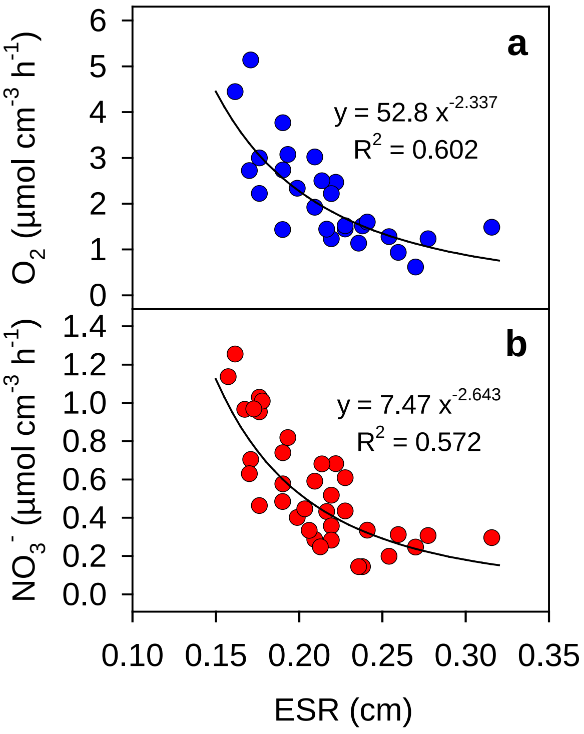

Figure S5: Volumetric consumption rates of (**a**) O_2 and (**b**) NO_3^- in sinking *Skeletonema* aggregates in relation to the equivalent spherical radius (ESR) of the aggregate. Total net consumption rates, measured across all ambient O_2 levels tested, are presented here as positive values.

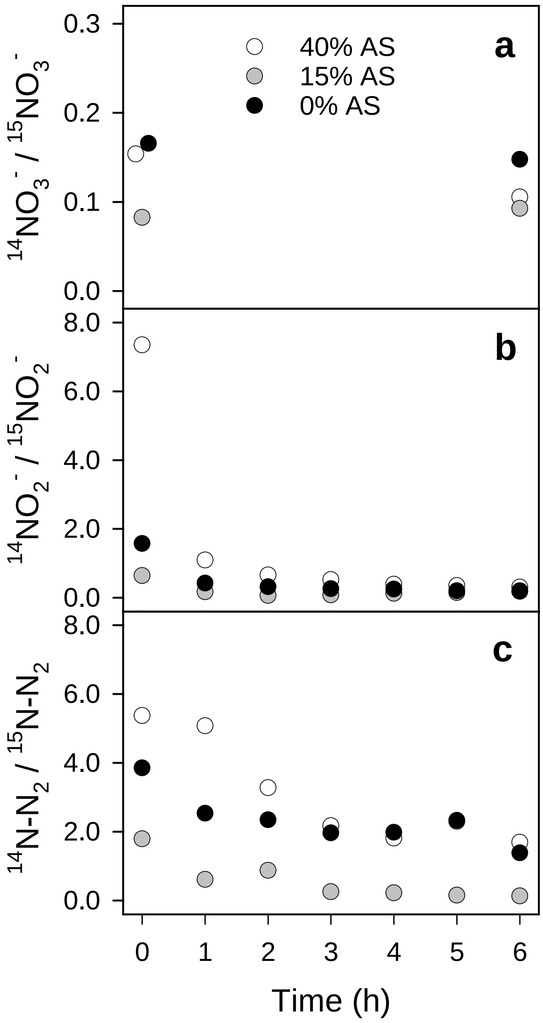

Figure S6: Concentration ratios of (**a**) $^{14}NO_3^-/^{15}NO_3^-$, (**b**) $^{14}NO_2^-/^{15}NO_2^-$, and (**c**) $^{14}N\text{-}N_2/^{15}N\text{-}N_2$ in incubations of sinking *Skeletonema* aggregates over time. Mean values of 5-9 aggregates incubated at different air-saturation levels (AS) are shown. Error bars are omitted for clarity.

Table S1: Effect of ambient O_2 level on N and O_2 turnover rates (data in Fig. 2): One-way ANOVA

				Homogeneous subgroups				
Reactant	*df*	*F*	*p*	0% AS	15% AS	40% AS	70% AS	100% AS
O_2	3,23,26	1.564	0.225	n.a.	A	A	A	A
NO_3^-	4,31,35	1.733	0.168	A	A	A	A	A
NO_2^-	4,31,35	20.985	0.000	A	B	BC	C	C
NH_4^+	4,31,35	4.890	0.004	AB	A	B	B	B
N_2O	4,26,30	14.225	0.000	A	A	A	B	B
N_2	4,31,35	5.960	0.001	A	A	AB	B	AB

Homogeneous subgroups (i.e., non-significantly different treatments according to Games-Howell's Post-Hoc test for non-homogeneous variances) share the same capital letter.
AS = air saturation; n.a. = not applicable

Table S2: Turnover rates different from zero (data in Fig. 2): One-sample *T*-tests

Reactant	0% AS	15% AS	40% AS	70% AS	100% AS
O_2	n.a.	$p < 0.001$	$p < 0.001$	$p < 0.001$	$p = 0.010$
NO_3^-	$p < 0.001$	$p < 0.001$	$p < 0.001$	$p = 0.002$	$p = 0.003$
NO_2^-	$p < 0.001$	$p = 0.004$	$p = 0.010$	$p = 0.381$	$p = 0.160$
NH_4^+	$p = 0.006$	$p = 0.009$	$p = 0.007$	$p = 0.010$	$p = 0.037$
N_2O	$p = 0.001$	$p = 0.001$	$p < 0.001$	$p = 0.001$	$p = 0.016$
N_2	$p = 0.002$	$p = 0.007$	$p = 0.030$	$p = 0.044$	$p = 0.232$

p-values < 0.05 indicate that turnover rates are significantly different from zero.
AS = air saturation; n.a. = not applicable
